# Enhancing Visual Perception and Motor Accuracy among School Children through a Mindfulness and Compassion Program

**DOI:** 10.3389/fpsyg.2017.00281

**Published:** 2017-02-24

**Authors:** Ricardo Tarrasch, Lilach Margalit-Shalom, Rony Berger

**Affiliations:** ^1^School of Education, Tel Aviv UniversityTel Aviv, Israel; ^2^Sagol School of Neuroscience, Tel Aviv UniversityTel Aviv, Israel; ^3^Faculty of Emergency Medicine, Ben-Gurion University of the NegevBeer-Sheva, Israel; ^4^PReparED Center for Emergency Response Research, Ben-Gurion University of the Negev, Beer-ShevaIsrael

**Keywords:** children, mindfulness, compassion, visual perception, motor accuracy, anxiety

## Abstract

The present study assessed the effects of the mindfulness/compassion cultivating program: “Call to Care-Israel” on the performance in visual perception (VP) and motor accuracy, as well as on anxiety levels and self-reported mindfulness among 4th and 5th grade students. One hundred and thirty-eight children participated in the program for 24 weekly sessions, while 78 children served as controls. Repeated measures ANOVA’s yielded significant interactions between time of measurement and group for VP, motor accuracy, reported mindfulness, and anxiety. *Post hoc* tests revealed significant improvements in the four aforementioned measures in the experimental group only. In addition, significant correlations were obtained between the improvement in motor accuracy and the reduction in anxiety and the increase in mindfulness. Since VP and motor accuracy are basic skills associated with quantifiable academic characteristics, such as reading and mathematical abilities, the results may suggest that mindfulness practice has the ability to improve academic achievements.

## Introduction

### Mindfulness Interventions for School-Children

During the last decade, there has been an increasing worldwide interest in the implementation of mindfulness based intervention programs for school-children (Waters et al., 2015). In these programs, students are exposed to techniques aimed to increase attention, social competence and emotional, cognitive and physical self-regulation (Meiklejohn et al., 2012). Albrecht et al. (2012) note in their review that these programs have the capacity to develop compassion, empathy and patience, improve the social climate within the school, increase student involvement in school activities and improve academic performance.

Mindfulness originates from Buddhist philosophy, but the practice has been secularized and adapted to Western society in programs such as Mindfulness-based stress reduction for adults (Praissman, 2008) and several programs for children (for a review, see: Meiklejohn et al., 2012). The most widely used definition of mindfulness is: “the awareness that emerges through paying attention on purpose, in the present moment, and non-judgmentally to the unfolding of experience moment by moment” (Kabat-Zinn, 2003, p. 145).

Numerous studies have been conducted on the effects of mindfulness on physical, cognitive and emotional measures among adults. Mindfulness has been found to reduce stress (For a review, see: Goyal et al., 2014), anxiety (For a review, see: Chen et al., 2012) and depression (Kumar et al., 2008; Hofmann et al., 2010), and increase well-being (For a review, see: Gu et al., 2015) and self-reported mindfulness (Cusens et al., 2010; Josefsson et al., 2011; Baer et al., 2012). The practice helps the practitioners to understand that they can remain focused on a task and ignore distractions, enabling them to reduce self-imposed burdens and impulsivity, whilst increasing their sense of personal well-being (Brown and Ryan, 2003; Carmody and Baer, 2008; Chiesa and Serretti, 2009). Mindfulness practice has also been shown to improve various cognitive skills, such as attention (Napoli et al., 2005; Semple et al., 2010; van de Weijer-Bergsma et al., 2012; Black and Fernando, 2014), memory (Jha et al., 2010; Mrazek et al., 2013), executive functions (Chan and Woollacott, 2007; Flook et al., 2010) and visuospatial processing (Zeidan et al., 2010).

The practice of mindfulness for children is similar to that for adults, but with shorter and more concrete exercises (Weare, 2013). Several recent reviews have shown its positive results (Black et al., 2009; Burke, 2009; Harnett and Dawe, 2012; Meiklejohn et al., 2012; Weare, 2013; Waters et al., 2015). Its effects among children and teenagers can be grouped into three areas: well-being, social competence and academic achievements. In terms of well-being, there is a large body of research supporting a positive impact including enhanced optimism (Broderick and Metz, 2009; Schonert-Reichl and Lawlor, 2010), reduced stress and anxiety (Beauchemin et al., 2008; Mendelson et al., 2010; Kuyken et al., 2013; van de Weijer-Bergsma et al., 2014) and increased ego-resilience (Huppert and Johnson, 2010). Support has also been obtained for enhanced social competence (Beauchemin et al., 2008; Schonert-Reichl and Lawlor, 2010), including more relaxed behavior, fewer social disturbances (Napoli et al., 2005; Semple et al., 2005; Bögels et al., 2008; Joyce et al., 2010; van de Weijer-Bergsma et al., 2012; Black and Fernando, 2014; Wisner, 2014) and decreased aggression (Schonert-Reichl and Lawlor, 2010). Zelazo and Lyons (2012) predict that mindfulness practice helps children in developing self-regulation and improving academic performance. Although, there is evidence for augmented self-regulation following mindfulness practice (Flook et al., 2015), there is still a lack of sufficient evidence concerning the effects of mindfulness practice on academic performance (Waters et al., 2015).

Improvements in academic abilities are difficult to observe as they may take a long time until they are reflected in academic achievement. Therefore, the assessment of indirect correlates or precursors may aid in evaluating the effects of mindfulness on academic performance. Several studies have shown that two key functions that serve as precursors to academic performance are visual perception (VP) and motor accuracy (Dhingra et al., 2010; Carlson et al., 2013; Son and Meisels, 2016).

### Visual Perception, Motor Accuracy, and Child Development

Visual perception refers to comprehending and organizing visual input from one’s environment (Sortor and Kulp, 2003). Studies have shown a positive correlation between VP and academic performance, including written expression achievement and mathematical skills (Kurdek and Sinclair, 2001; Assel et al., 2003; Dhingra et al., 2010; Carlson et al., 2013).

Motor accuracy is the ability to perform an activity in a continuous manner, whilst maintaining movement accuracy and efficiency, executing the minimal amount of movement required for achieving a certain goal. Several studies have shown a significant correlation between motor accuracy and handwriting quality in children (Cornhill and Case-Smith, 1996; Karlsdottir and Stefansson, 2002; Kaiser et al., 2009). There is a significant correlation between motor accuracy and current mathematical and written expression achievements and later reading, math and science scores (Sortor and Kulp, 2003; Grissmer et al., 2010; Carlson et al., 2013; Son and Meisels, 2016).

In addition, these two functions and their combined incorporation as visual-motor integration stand on their own as key elements in child development. Visual-motor integration has been described as being multifaceted and encompassing: visual receptive functions; visual cognitive functions; fine motor ability; and the integration of visual, cognitive, and motor processes (Dankert et al., 2003; Schneck, 2010). Accordingly, improving visual-motor skills is one of the main objectives of occupational therapists who work with preschool and early elementary school children (Ratzon et al., 2007).

How does mindfulness practice relate to VP, motor accuracy and visual-motor integration? According to Relational Frame Theory (RFT; Hayes et al., 2001), human behavior is largely governed by linguistic and cognitive networks of mutual relations (i.e., relational frames) that allow us to learn without direct experience. In other words, RFT suggests that we learn to respond to an event based on its ascribed relation to another event or based on social conventions rather than its physical properties. This way of dealing with the external world hinders our VP as we tend to be distracted by our learned relational frames. The practice of mindfulness enables us to gain control over attention (Malinowski, 2013; Posner et al., 2015), and to perceive objects as directly experienced stimuli rather than abstract concepts (i.e., relational frames). With generally increasing awareness, mediation can therefore influence both the quality (accuracy) and quantity (detection) of perception (Tloczynski et al., 2000). Indeed, several studies have found that mindfulness practice leads to improvements in selective attention (Moore and Malinowski, 2009; Jensen et al., 2012), which in turn has been shown to be a key mechanism of VP (Luck and Ford, 1998; Hodgins and Adair, 2010). Additionally, studies have demonstrated the benefits of meditation on VP, including improvement in stimulus detection (Brown et al., 1984; Tloczynski et al., 2000; Jha et al., 2007; Hodgins and Adair, 2010).

One of the core elements of motor accuracy is increased perceptual-motor awareness. Neuroimaging and neurophysiological studies assessing the neural basis of perceptual-motor awareness have found that the main brain areas involved in this process are the cerebellum (Blakemore et al., 2001), the parietal cortex (Sirigu et al., 2004), the angular gyrus (Farrer et al., 2004), the insular cortex (Farrer et al., 2004; Karnath, 2005), and the prefrontal cortex (Slachevsky et al., 2003). As mentioned above, mindfulness meditation is aimed at expanding the attention to all available inputs (sensory, bodily, or mental) within consciousness in the present moment. Practitioners are trained in the conscious execution and continuous awareness of body movements. Mindfulness practice has been shown to activate a neural network comprising the lateral prefrontal cortex, insula, secondary somatosensory cortex and inferior parietal cortex (Farb et al., 2007). This network overlaps with the aforementioned brain areas known to be activated in relation to the experience of self-agency and perceptual-motor awareness. Thus, mindfulness meditation practice is expected to improve motor accuracy. Preliminary support for this prediction was provided by a recent study that found that adults practicing mindfulness show better motor accuracy (Naranjo and Schmidt, 2012).

### Study Aims

The aim of this study is to explore the impact of mindfulness practice on VP and motor accuracy in children. For this purpose, this study implements the “Call to Care- Israel” (C2C-I), a mindfulness and compassion based program that utilizes developmentally appropriate contemplative practices and social emotional-emotional skills. To the best of our knowledge, no studies have been performed on the effects of mindfulness practice on VP and motor accuracy among children. We hypothesize that children participating in the C2C-I intervention, but not in the control group, will exhibit improvements in VP, motor accuracy, and mindfulness as well as reduction in their anxiety levels. In addition, we hypothesize that improvements in VP and motor accuracy will correlate with anxiety reduction and increase in mindfulness.

## Materials and Methods

### Participants

The study was conducted with 216 4th and 5th grade Jewish-Israeli students, 138 in the Call to Care-Israel (C2C-I) intervention and 78 in a control group. Children were recruited from three different schools in central Israel, with a similar middle class socio-economic background. The experimental group included 71 girls and 67 boys (age range 9.4–11.3, average 10.25, standard deviation 0.6), and the control group 36 girls and 42 boys (age range 9.5–11.5, average 10.6, standard deviation 0.6).

### Procedure

Three schools, that were selected for the study expressed interest in implementing the mindfulness and compassion cultivating program. One school agreed to randomly implement the C2C-I program in two classes (*n* = 53), while another served as a wait-list control group (*n* = 21). Among the other two schools, the C2C-I program was implemented in one school (*n* = 85), while the other served as a wait-list control group (*n* = 57). The C2C-I program was presented to the parents whose children participated in the program, enlisting their support and cooperation particularly with the students’ homework assignments. Thereafter, the program was presented to the teachers in the schools whose students participated in the intervention.

In the first phase of the experiment, children individually performed the VP test, guided by one of two optional experimenters. They then performed the motor coordination (MC) test in their classroom, while required to work as quietly as possible, without interfering with the work of their classmates. In a separate session, children filled-out the questionnaires while in the classroom. In the second phase, the C2C-I meetings were held in the experimental group. The third phase was held during the week after the termination of the program, and was similar to the first phase.

The study was reviewed and approved by the ethics committee for human subjects at Tel Aviv University and approved by the chief scientist of the Israeli Ministry of Education. Participants’ parents provided signed consent for their children’s participation in the study, and children were told that they could cease their participation in the study at any time. No children participated in the study without parental consent. Parents were given contact information to obtain further details about the study.

### Intervention

The “Call to Care-Israel” for children is a school-based program that was written by a team of Israeli experts in collaboration with the Mind and Life Institute (Charlottesville, VA, USA). It is based on a compassion-cultivating program that was developed in the Mind and Life Institute (Dodson-Lavelle et al., 2015, Unpublished) and on the Enhancing Resiliency Among Students Experiencing Stress (ERASE-S) program, a social-emotional program that has proven its efficacy in reducing students’ stress and anxiety and improving their level of functioning at school (Berger et al., 2007, 2012; Berger and Gelkopf, 2009). The main goal of the program is to help children develop mindfulness skills and to cultivate a caring and compassionate school climate between the students, teachers and parents as well as to promote academic achievement and foster ethical behavior.

Students attended 24 weekly meetings of the C2C-I program during 7 months. The meetings were 45 min long, and were administered by facilitators with years of mindfulness practice, experience in working with children, and trained to administer the C2C-I program. The homeroom teachers were present during the meetings but served as observers rather than active facilitators.

The sessions were divided into three modes: receiving care, developing self-care and extending care. In the receiving care mode, the sessions focused on helping the students understand the universal need for care, explore their difficulties in receiving care, and teach them to reach for others when needed. The core practices and skills developed in this mode included basic mindfulness skills (i.e., focus on breath, body scan, mindful eating and walking), learning to re-experience moments of interconnection and warmth and inner safety.

The self-care mode sessions focused on helping students develop an awareness of their needs and their barriers for self-care. Students were taught self-soothing techniques and strategies for how to deal with stressful experiences. Core practices and skills in this mode included mindfulness and contemplation with a focus on soothing care-figures, re-experiencing comforting situations, relaxation strategies (i.e., muscular relaxation and safe place imagery), and stress-management skills (i.e., stress-inoculation, self-affirmation, and self-talk).

Finally, the sessions in the extending care mode aimed to direct care toward others including friends, children they do not know, and even children they dislike (e.g., find annoying or mean), to develop awareness of constricting thoughts, such as stereotypes and prejudices, and to expand empathy and care across in-group/out-group boundaries. The core practices and skills in this mode included developing a compassionate mindset, practicing loving kindness meditation, and developing perspective-taking and empathy skills.

Each session in the three modes included: psychoeducational materials (e.g., age-appropriate presentations of the brain correlates of mindfulness and compassion); contemplative practices (e.g., teaching mindful breath counting, body scan, or caring-figure meditation); social-emotional skills (e.g., identifying and sharing emotions, learning to receive and give social support, or developing perspective-taking and empathy skills); group activities (e.g., sharing positive and negative feelings with peers or role-playing difficult situations); and homework assignments (e.g., interviewing family members regarding their ideas about care and compassion or teaching and practicing with them the learned skills). Following each session, the facilitators used the school web site to teach parents about the concepts that were introduced to their children and to explain the program-related homework assignments. Parents were encouraged to work supportively with their children, particularly when the home assignment required their participation. Additionally, students were also given mindfulness dairies in which they were encouraged to document their feelings and thoughts in school, at home, and in the community, as well as their experiences with the contemplative practices and the social-emotional skills.

### Measures

The study used the Beery-Buktenica Developmental Test of Visual-Motor Integration (VMI), sixth edition, which has been found to be independent of culture and sex (Beery and Buktenica, 1997). The Beery-VMI is a norm-referenced measure that was developed and standardized through studies involving over 12,500 children (Beery et al., 2010). It was developed as an early screening tool to identify children who have not fully integrated their visual and motor abilities. The adolescence and adult versions were developed subsequently. The pediatric version of the Beery-VMI is for children/adolescence 2–18 years 11 months and includes a sequence of geometric shapes, arranged in a developmental sequence, to be identified copied or imitated by children using pencil and paper. The Beery-VMI is a widely used visual motor assessment because of its extensive and well-documented psychometric properties (Beery et al., 2010).

The test includes three parts: visual-motor integration, VP and MC. This study used only the last two measures. The test’s norms allow calculating for each child a population percentile based on his or her age.

In the VP test, children were presented with 27 geometric forms. For each form, an identical form had to be chosen among others that looked nearly but not exactly the same. The child had to point to the identical figure, so motor requirements were minimal. The test ended after three consecutive errors or at a time limit of 3 min. The number of correct identified forms was recorded and converted to a relative percentile score, based on VMI sixth edition norms. The internal reliability of the test is α = 0.85, and the test–retest reliability is *r* = 0.85. The inter-rater reliability is *r* = 0.98.

The MC test assesses motor accuracy. Children had to trace the same 27 geometric forms, that become progressively more complex and challenging, with a pencil within 5 min, with the purpose of staying within double-lined paths surrounding the shape. This task requires delicate motor control over the hand, especially when the shapes are small, or the distance between the lines is small. The subject has to anticipate the changes in the direction of the line, and plan when to slow down or stop their hand movement. Drawing the line demands precision, as required in writing tasks (Kaiser et al., 2009). The participating children received one point for each correctly completed shape. The obtained points were converted to a relative percentile score, based on VMI sixth edition norms. The internal reliability of the test is α = 0.87, and the test–retest reliability is *r* = 0.86. The inter-rater reliability is *r* = 0.93 (Beery and Buktenica, 1997).

A shortened version of the Spence Children’s Anxiety Scale (SCAS) (Spence, 1998), was also used in the study. It includes eight items to be ranked on a four-point Likert Scale ranging from 1 (never) to 4 (always), with items such as “I worry that something bad will happen to me.” The score of the test is the average of its items. The internal reliability in our study was α = 0.79 in the pre-test and α = 0.83 in the post-test.

The study also included the Five Facet Mindfulness Questionnaire (FFMQ), developed by Baer et al. (2006). The FFMQ is a self-report instrument that assesses an individual’s tendency to be mindful in everyday life (Baer et al., 2006). It contains 39 items in five subscales: the tendency to notice inner experiences (observing), the tendency to be able to label one’s inner experiences (describing), attending to activities and experiences in the moment (acting with awareness), taking a non-evaluative or non-judgmental stance toward thoughts and feelings (non-judging) and non-reactivity to inner experience (non-reacting). Items are scored on a five point Likert scale, ranging from 1 (never or rarely true) to 5 (very often or always true). The final score can be calculated as FFMQ-total, by averaging all questions, or separated out into subscales. All subscales and total-FFMQ were used in this study. The FFMQ exhibits excellent psychometric properties (see Baer et al., 2006). In our study, the internal reliabilities of the scales in the pre- and post-measures were: general score α = 0.86 and α = 0.88; observing α = 0.65 and α = 0.66; describing α = 0.78 and α = 0.77; acting with awareness α = 0.68 and α = 0.75; non-judging α = 0.81 and α = 0.78; and non-reacting α = 0.79 and α = 0.73.

### Statistical Analyses

*T*-tests for independent samples were used to compare between the two groups in the pre-measures. In order to assess the effects of the program on VP, MC, anxiety and reported mindfulness, repeated measures ANOVAs were performed, comparing the performance of the control and C2C-I groups, before and after the program. In order to control for possible differences between boys and girls, gender was used as an additional independent variable. These 2 × 2 × (2) ANOVA’s with the within-subjects effects of time of measurement (before and after the program) and between-subjects factors of group (control, C2C-I) and gender, were performed separately for each of the dependent measures used in the study. Partial eta-squared values were computed as measures of effect size. Significant effects were followed by Tukey Honest Significant Difference (HSD) *post hoc* comparisons. In order to assess whether the results of the students in the school where the mindfulness program was randomly assigned differed from the results of the students in the other schools, separate repeated measures ANOVAs were performed for controls and C2C-I groups, comparing between randomly assigned and non-randomly assigned schools. In order to assess whether the improvements in VP and motor accuracy were concomitant between each other and with anxiety reduction and mindfulness increase, Pearson correlations were calculated between difference scores that were calculated by subtracting post- from pre-scores.

Some children were not present at the post-test, and were eliminated from the specific analyses where their data was missing. Fourteen children had missing data in the VP test; six in the control group and eight in the C2C-I group. Twenty children had missing data in the MC test; eight in the control group. Four children had missing data in the anxiety and mindfulness questionnaires; three in the control group.

## Results

No differences were obtained between the control and C2C-I groups before the intervention in VP [*t*(200) = 1.13, *p* = 0.26], MC [*t*(194) = 0.87, *p* = 0.38], anxiety [*t*(210) = 0.48, *p* = 0.63] and mindfulness factors [General Score *t*(210) = 0.76, *p* = 0.45]; Observing [*t*(210) = -1.69, *p* = 0.09]; Describing [*t*(210) = -0.71, *p* = 0.48]; Acting with awareness [*t*(210) = -0.31, *p* = 0.76]; Non-judging [*t*(210) = 0.53, *p* = 0.59]; and Non-Reacting [*t*(210) = -0.42, *p* = 0.68].

### Visual Perception

**FIGURE 1:**
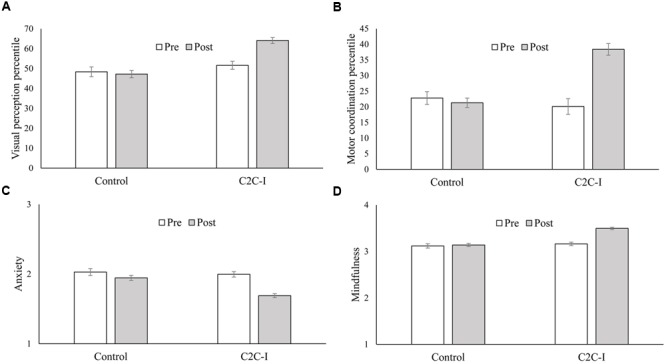
**Two-way interaction between time and group on visual perception percentile (A)**, motor coordination percentile **(B)**, anxiety **(C)** and mindfulness **(D)**.

A significant main effect of gender was obtained [*F*(1,198) = 3.96, *p* < 0.05, $\eta_{p}^{2}$ = 0.020]. Girls had a better VP than boys. In addition, significant main effects of group [*F*(1,198) = 19.04, *p* < 0.001, $\eta_{p}^{2}$ = 0.088], time [*F*(1,198) = 12.57, *p* < 0.001, $\eta_{p}^{2}$ = 0.060] and interaction between group and time [*F*(1,198) = 17.95, *p* < 0.001, $\eta_{p}^{2}$ = 0.083] were obtained. As can be seen in **Figure 1A**, Tukey’s HSD revealed a significant improvement in VP in the C2C-I group (*p* < 0.01), but not in the control group.

### Motor Coordination

A significant main effect of gender was obtained [*F*(1,192) = 17.38, *p* < 0.001, $\eta_{p}^{2}$ = 0.083]. Girls showed better MC as compared to boys. In addition, significant main effects of group [*F*(1,192) = 8.61, *p* < 0.005, $\eta_{p}^{2}$ = 0.043], time [*F*(1,192) = 32.92, *p* < 0.001, $\eta_{p}^{2}$ = 0.146] and interaction between group and time were obtained [*F*(1,192) = 46.04, *p* < 0.001, $\eta_{p}^{2}$ = 0.193]. As can be seen in **Figure 1B**, Tukey’s HSD revealed a significant improvement in MC in the C2C-I group (*p* < 0.01), but not in the control group.

### Anxiety

Significant main effects of group [*F*(1,208) = 7.64, *p* < 0.01, $\eta_{p}^{2}$ = 0.035], time [*F*(1,208) = 95.90, *p* < 0.001, $\eta_{p}^{2}$ = 0.316] and interaction between group and time were obtained [*F*(1,208) = 31.08, *p* < 0.001, $\eta_{p}^{2}$ = 0.130]. As can be seen in **Figure 1C**, Tukey’s HSD revealed a significant reduction in anxiety in the C2C-I group (*p* < 0.01), but not in the control group.

### Mindfulness

The effects of the repeated measures ANOVA’s are presented in **Table 1**. No significant effect of gender nor interactions between time, group and gender were obtained, therefore these effects are not presented. As can be seen, the interaction between group and time was significant for all factors. Tukey’s HSD revealed a significant increase in the mindfulness measures in the C2C-I group (*p* < 0.001), but not in the control group. **Figure 1D** presents the interaction for the general mindfulness score.

**Table 1:** Summary of the group, time and group by time effects obtained in the repeated measurements ANOVA’s performed on the mindfulness scores.

Score	Effect of group *F*(1,208)=	Effect of time *F*(1,208)=	Interaction between group and time *F*(1,208)=
General score	16.45, *p* < 0.001	117.71, *p* < 0.001	91.22, *p* < 0.001
	$\eta_{p}^{2}$ = 0.073	$\eta_{p}^{2}$ = 0.361	$\eta_{p}^{2}$ = 0.305
Observing	17.27, *p* < 0.001	65.48, *p* < 0.001	46.92, *p* < 0.001
Describing	3.94, *p* < 0.05	4.71, *p* < 0.05	19.91, *p* < 0.001
Acting with awareness	3.46, *p* = 0.06	29.32, *p* < 0.001	31.16, *p* < 0.001
Non-judging	4.39, *p* < 0.05	96.95, *p* < 0.001	45.71, *p* < 0.001
Non-reacting	14.06, *p* < 0.001	82.79, *p* < 0.001	58.01, *p* < 0.001

No significant interactions between time and school were obtained in any of the measures used, neither in the control nor in the C2C-I groups (all *p*’s > 0.14). In other words, no significant differences were obtained between students in the school where the mindfulness program was randomly assigned and students in the other schools.

A significant correlation was obtained between the improvement in MC and the reduction in anxiety (*r* = -0.15, *p* = 0.04), and the improvements in the general mindfulness (*r* = 0.24, *p* < 0.001), observing (*r* = 0.23, *p* = 0.002), non-judging (*r* = 0.18, *p* = 0.01), and non-reacting (*r* = 0.21, *p* = 0.004) scores. No significant correlations were obtained between improvements in VP and improvements in MC, anxiety or mindfulness.

## Discussion

### Major Findings and Comparison to Previous Research

The present study assessed the effects of a mindfulness and compassion program on the performance in VP and motor accuracy, as well as on anxiety levels and self-reported mindfulness among 4th and 5th grades students. The results indicated that students who participated in the C2C-I group preformed significantly better on both tasks than students in the wait-list control group. The results also showed a significant increase in students’ mindfulness and a reduction in their anxiety levels. We suggest that, as predicted by the RFT theory, the increase in students’ mindfulness, namely, attending to stimuli in the present and suspending the cognitive processing, improved their motor accuracy. This suggestion is in line with the correlation obtained between the improvements in MC and mindfulness scores. Our findings are in concordance with studies that evaluated the effects of mindfulness on VP in adults, and found that training in mindfulness improved target recognition (Brown et al., 1984), and that experienced meditators showed better performance in a visual task as compared to inexperienced meditators (Tloczynski et al., 2000). In addition, meditation was found to be linked with more efficient and flexible VP (Hodgins and Adair, 2010).

The improvement in motor accuracy and VP may stem from the fact that mindfulness practice helps the practitioner become more mindful of all information, internal and external, at a given moment. The skill of becoming more mindful is expressed, not only in the course of one’s practice, but is carried over to daily activities, such as eating, walking and becoming more aware of the movements of the body. Accordingly, the practice of mindfulness leads the practitioners to slow down their actions when faced with movement-related tasks. This in turn improves their motor control and accuracy, through top-down processes, while allowing them to better assess, at any given point, the quality of their performance, and to make corrections. Similar findings were also provided by Naranjo and Schmidt (2012), who explored the effects of mindfulness practice on motor accuracy in adults, and found that subjects who practiced mindfulness exhibited significantly less errors in motor accuracy compared to controls.

Visual perception and motor accuracy are basic skills associated with quantifiable academic domains, such as reading and mathematical abilities (Sortor and Kulp, 2003). Thus, should the findings of our study be replicated, mindfulness practice has the potential to be a supplemental strategy that facilitates children potential to improve academic achievements, specifically in the areas of reading and mathematics. These skills are especially crucial among the population of kindergarten children, as part of their ongoing preparation for their school years to come, and among students with reading problems or learning disabilities. Future studies should explore this possibility, and specifically evaluate the impact of mindfulness practice in these populations.

In terms of anxiety, a significant reduction in reported anxiety was observed in the mindfulness group only. This finding is consistent with other studies that found reductions in anxiety after mindfulness practice, among adults (Baer, 2003; Roemer et al., 2008; Hofmann et al., 2010; Kim et al., 2010), adolescents (Beauchemin et al., 2008; Zylowska et al., 2008; Biegel and Brown, 2009; Wisner, 2014) and children (Napoli et al., 2005; Mendelson et al., 2010; Semple et al., 2010; Klatt et al., 2013). The reduction of anxiety found among our students may be attributed to the improved performance in motor accuracy as children may have been more attuned to stimuli and better able to process information. Alternatively, the improvements in motor accuracy could be attributed to reductions in anxiety, as less anxious children are likely to become more focused and attuned, and consequently improve their performance in class (Weare, 2013). This is also in line with the Liverpool mindfulness model (Malinowski, 2013) which assumes that mindfulness practice affects emotional and cognitive flexibility, and in turn improve behavior. In the same vein, Zeidan et al. (2010) suggest that mindfulness practice reduces anxiety and its concomitant negative thoughts and mind wandering, thus improving one’s ability to process information and leading to improved cognitive performance. This explanation does not seem to fit our results in relation to VP, as no correlation between improvements in VP and anxiety reduction were obtained. Furthermore, the lack of a significant correlation between the improvements in VP and motor accuracy, supports a dissociation between these two processes (Sirigu and Duhamel, 2001), and suggests differential underlying mechanisms. Future studies should explore the specific components of the mindfulness process that account for changes in VP.

The C2C-I group showed a significant improvement in the general mindfulness score, as well as in all five mindfulness factors. This finding is consistent with those in many other studies, among adults and children, showing an increase in reported mindfulness following mindfulness practice (e.g., Shapiro et al., 2008; Schonert-Reichl et al., 2015). Hayes and Feldman (2004) claim that in the course of mindfulness practice, the practitioners attains a non-judgmental state of mind which allows them a sense of distance from the experience itself. As a result, there is an increase in one’s ability to re-focus to the task at hand, thus reducing automatic reactions to irrelevant stimuli. It is plausible that in our study, subjects in the mindfulness group improved their motor accuracy abilities through the same mechanisms (i.e., being more attuned to the ‘here and now,’ more aware of internal and external interferences, not acting in an impulsive manner, and working in an accurate and composed fashion).

In sum, our results suggest that the implementation of a mindfulness and compassion program among young children can enhance their VP and motor accuracy, and consequently increase their chances to attain good academic performance. This may be especially relevant for children with low academic performance, and perhaps for children with specific learning disabilities.

### Strengths, Limitations, and Recommendations for Future Studies

Strengths of the present study included an emphasis on investigating a large sample, matching a control group (in terms of age and socio-economic status), and using, not only subjective, but also objective measures. Furthermore, we used a much longer intervention compared to most studies conducted among children. Our intervention lasted 7 months, addressing the need for longer interventions as suggested by Meiklejohn et al. (2012).

Despite the promising results of this study, they should be interpreted in light of the following limitations. First, our study included the use of a passive control thus it is possible that the improvements in VP and motor accuracy were due to demand expectations. Second, a lack of a full randomization (only one of the participating schools was randomized by class) may have resulted in a sample bias. However, the lack of interaction between school (randomized vs. non-randomized) and time of measurement, in neither of the experimental groups, suggests that such bias did not influence our results. Third, our sample was recruited from three schools whose principals had previously expressed interest in implementing a mindfulness and compassion cultivating program. Therefore, it is possible that the schools’ administrators were especially invested in the successful implementation of the program, whereas principals who are less inclined to run such programs might not be as vigilant about implementing the program with fidelity. As a result, the findings may not generalize to settings in which the school administration is not as equally dedicated to the practice. Future studies should consider the possibility of including an active control group, implement the intervention in a number of schools with a better randomization strategy, as well as to evaluate the impact of the program, directly on school performance (i.e., GPA scores).

## Conclusion

Our results are encouraging, as they suggest that mindfulness can be used as an additional tool to indirectly increase children’s VP and motor accuracy and potentially improve their academic performance. Accordingly, we would recommend training teachers, counselors, school psychologists, and occupational therapists with mindfulness tools during their studies, so that they may implement these techniques in their work with children.

## Author Contributions

RT and RB planned the study and supervised its performance. RT performed the statistics and wrote the MS. RB helped with the writing. LM-S was in charge of logistics, administered the tests, and helped in the writing.

## Conflict of Interest Statement

The authors declare that the research was conducted in the absence of any commercial or financial relationships that could be construed as a potential conflict of interest.
